# A comprehensive analysis of copy number variations in diverse apple populations

**DOI:** 10.1186/s12864-023-09347-9

**Published:** 2023-05-11

**Authors:** Jinsheng Xu, Weihan Zhang, Ping Zhang, Weicheng Sun, Yuepeng Han, Li Li

**Affiliations:** 1 Hubei Key Laboratory of Agricultural Bioinformatics, College of Informatics, Huazhong Agricultural University, Wuhan, 430070 China; 2 CAS Key Laboratory of Plant Germplasm Enhancement and Specialty Agriculture, Wuhan Botanical Garden, The Innovative Academy of Seed Design, Chinese Academy of Sciences, Wuhan, 430074 China; 3 Hubei Hongshan Laboratory, Huazhong Agricultural University, Wuhan, 430070 China

**Keywords:** Apple, Copy number variation, Population differentiation, Defense response

## Abstract

**Background:**

As an important source of genetic variation, copy number variation (CNV) can alter the dosage of DNA segments, which in turn may affect gene expression level and phenotype. However, our knowledge of CNV in apple is still limited. Here, we obtained high-confidence CNVs and investigated their functional impact based on genome resequencing data of two apple populations, cultivars and wild relatives.

**Results:**

In this study, we identified 914,610 CNVs comprising 14,839 CNV regions (CNVRs) from 346 apple accessions, including 289 cultivars and 57 wild relatives. CNVRs summed to 71.19 Mb, accounting for 10.03% of the apple genome. Under the low linkage disequilibrium (LD) with nearby SNPs, they could also accurately reflect the population structure of apple independent of SNPs. Furthermore, A total of 3,621 genes were covered by CNVRs and functionally involved in biological processes such as defense response, reproduction and metabolic processes. In addition, the population differentiation index (${V}_{st}$) analysis between cultivars and wild relatives revealed 127 CN-differentiated genes, which may contribute to trait differences in these two populations.

**Conclusions:**

This study was based on identification of CNVs from 346 diverse apple accessions, which to our knowledge was the largest dataset for CNV analysis in apple. Our work presented the first comprehensive CNV map and provided valuable resources for understanding genomic variations in apple.

**Supplementary Information:**

The online version contains supplementary material available at 10.1186/s12864-023-09347-9

## Background

Apple is an economically important fruit tree and has been widely grown in temperate regions around the world. The cultivated apple (*Malus* × *domestica* Borkh.) has been domesticated from the wild apple *Malus sieversii* and hybridized with *Malus sylvestris* [1, 2]. Differences of phenotypes between cultivated apples and wild relatives are enormous in terms of fruit size, taste and other agronomic traits [3–5]. Genetic sources of trait variation can be mainly attributed to single nucleotide polymorphism (SNP) and copy number variation (CNV)[6]. However, to date, studies that revealed molecular mechanisms related to important traits in apple were focused on SNPs, leaving the information of CNV largely unexplored. As an unbalanced structural variation, CNV is defined as deletions and duplications longer than 50 bp in length [7–9]. CNV can alter the dosage of DNA segments, leading to changes of copy numbers in different individuals, which in turn may affect gene expression level and phenotypic variation [10, 11]. Compared with SNPs, the number of CNV is much less in the genome, but total number of base pairs impacted by CNV are significantly higher than that of the SNPs [12]. Therefore, CNV is an important source of genetic variation that could fill the missing links in the population genetics.

Several studies focused on CNVs revealed that they are ubiquitous widely spread among plant genomes and might be likely to be associated with disease resistance and stress responses such as submergence tolerance and anaerobic germination tolerance in rice [13, 14], nematode resistance in soybean [15], aluminum tolerance and resistance to Goss’s Wilt in maize [16, 17] and frost tolerance in wheat [17]. In addition, CNVs were also shown to be related to diverse trait variation. In wheat, an increased copy number of *Ppd-B1* and *Vrn-A1* is associated with altered flowering time [18]. In rice, a tandem duplication of *GL7* and partial deletion of *GSE5* contribute to grain size in rice [19, 20]. In cucumber, a 30.2 kb duplication defining the *F* locus gives rise to gynoecy [21], while a 1.4 kb deletion of *CSR-D* results in changes of fruit weight in tomato [22]. These studies uncovered the important roles CNV plays in adaption, resistance and development and it is an indispensable part of genomic variations.

The array comparative genomic hybridization (aCGH) and SNP arrays have been used for CNV detection in the last few years. With the development of sequencing technology, next-generation sequencing (NGS) is becoming popular for its high throughput and increasingly competitive cost [23]. There are four types methods for CNV detection based on NGS data: read-pair (RP) method utilizing the position mapped to the reference genome; split-read (SR) method relying on the aberrant mapping of the pair-end reads; read-depth (RD) method depending on the normalized depth of reads; de novo assembly method which is used to refine the identification of CNV breakpoints and infer structure of CNV [6]. Each method has its own strengths and shortcomings in terms of accuracy and precision. A typical strategy is to combine multiple methods to minimize false positives [24–26]. For example, a comprehensive AthCNV dataset was identified using a benchmarking pipeline that combined three methods (RP, SR and RD) in Arabidopsis [27]. SpeedSeq, a powerful tool designed for CNV identification, combined RP, SR and RD methods to improve detection efficiency and has been widely used in whole-genome CNV analysis [26, 28].

CNV plays a vital role in apple fruit development. It was reported that a tandem duplication in the upstream regulatory region of *MdMYB10* and a 930 bp region upstream of *MdMYB110a* (a paralog of *MdMYB10*) were responsible for red-fruit-flesh phenotypes in apple [29, 30]. To the best of our knowledge, there is only one study of NGS-based genome-wide CNV in apple, which used data of 30 cultivated apple accessions and identified 876 CNVRs enriched in resistance (R) gene models [31]. There is no genome-wide study of CNVs by comparing wild and cultivated apple at population level. Given a large amount of genomic re-sequencing data of apple released recently [4, 32], we collected data of 346 accessions (289 cultivars and 57 wild relatives) and identified CNVs based on GDDH13 reference genome. Subsequently, we explored population genetic characteristics of CNVs and further investigated their underlying impacts by analyzing GO enrichment pattern and copy number differentiated genes.

## Results

### CNV calling procedure and simulation evaluation

**Fig. 1:**
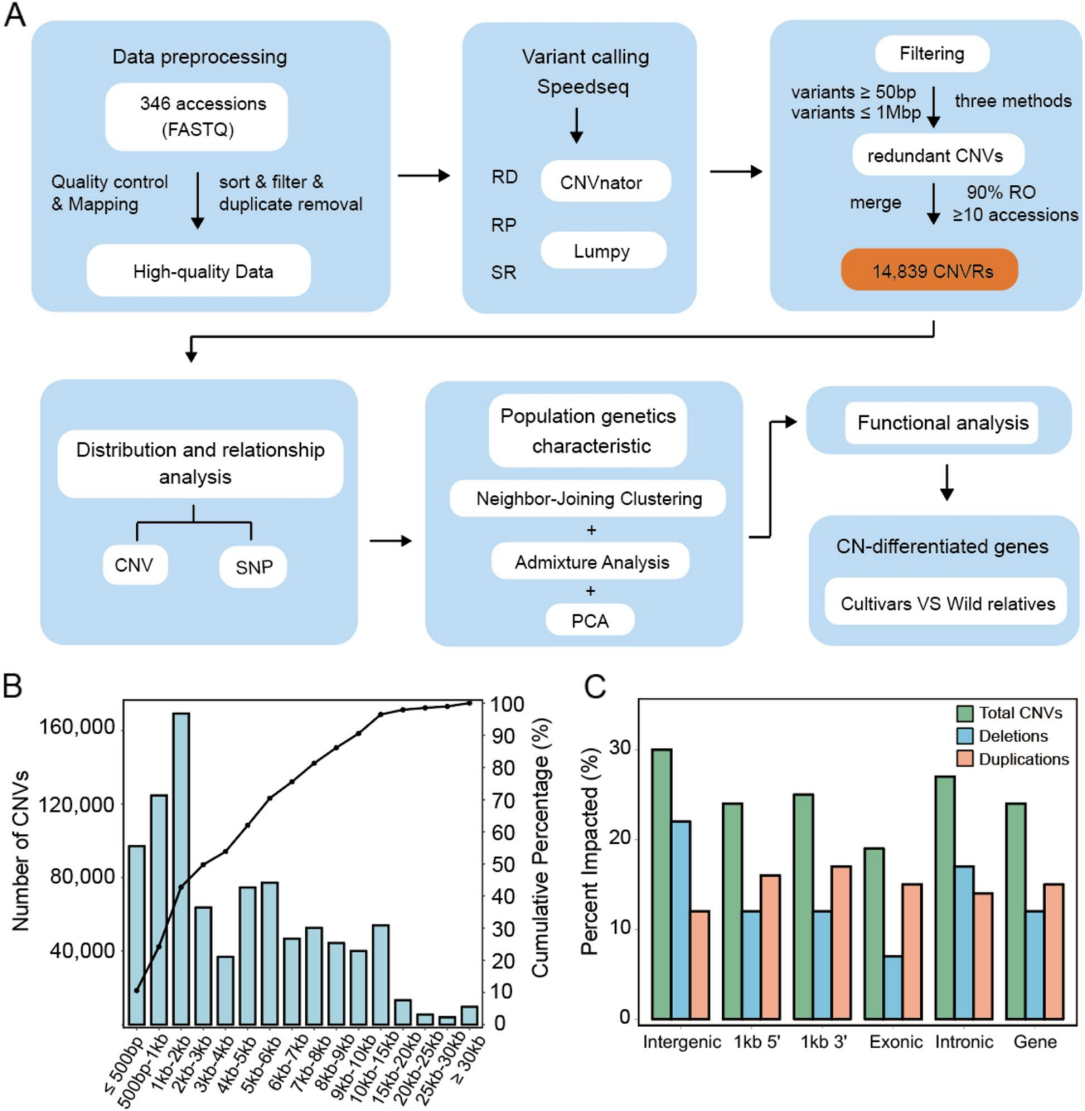
Summary statistics of CNVs in apples. **A** The whole CNV analysis workflow. **B** CNV size interval distribution. **C** CNV representation within apple genome features. The percentage of sequence classes impacted by duplications and deletions

CNV identification in apple was performed as illustrated in the pipeline (Fig. 1A). Briefly, a strategy that integrates RP, SR and RD methods was used. A total of 1.56 Tb high-quality whole genome sequencing data of 346 accessions were compiled for analysis (Additional file 2: Table S1). After aligning clean reads to the reference genome 'GDDH13 Version 1.1', the average depth was ~ 7.2X and genome coverage was ~ 92%, which revealed that it was sufficient for CNV detection [33, 34].

To ensure the accuracy of CNV detection, we evaluated the performance of Speedseq using simulated CNV in the apple genome (see Methods). The results showed that it was able to detect simulated CNV at sequencing depth levels commonly used in genomics studies (Additional file 1: Fig. S1). For sequencing depth between 5 and 10X which majority of our data fall in, the method we used can obtain true positive rate (TPR) of ~ 65% and ~ 75% for deletions and duplications, respectively. With the increase of sequencing depth, the improvement of TPR becomes marginal and saturates at ~ 84% and ~ 80% for deletions and duplications, respectively.

### Basic summary of CNV

A total of 914,610 CNVs (838,642 deletions and 75,968 duplications) were identified from 346 accessions (Fig. 1B; Additional file 2: Table S2) using GDDH13 reference genome. The mean number of CNVs per accession was 2,643 (ranging from 910 to 7,272) with a mean of 2,423 deletions and 220 duplications. To confirm reliability of identified CNVs, manual check on the reads mappings was performed. Here, a 630 bp deletion in the 5’UTR of *MdCBF2* and a duplication covering *MD15G139100* were selected as exemplary regions to show reliability of the identified CNVs (Additional file 1: Fig. S2).

To evaluate the influence of CNVs on genomic features, the proportion of bases in all non-overlapping CNVs was calculated. Among all the features, exonic regions showed the lowest percentages of CNV (19.86%). CNV was reduced in gene bodies (23.86%) compared with intergenic regions (29.59%) (Fig. 1C; Additional file 2: Table S3). On the flip side, deletion rates were lower in coding sequences (7.54%) than flanking sequences (24.62%). Additionally, the distribution of duplications along the whole genome (11.65% ~ 16.75%) seemed to be more even than deletions (7.54 ~ 21.88%).

### CNVR distribution and linkage analysis with SNPs

**Fig. 2:**
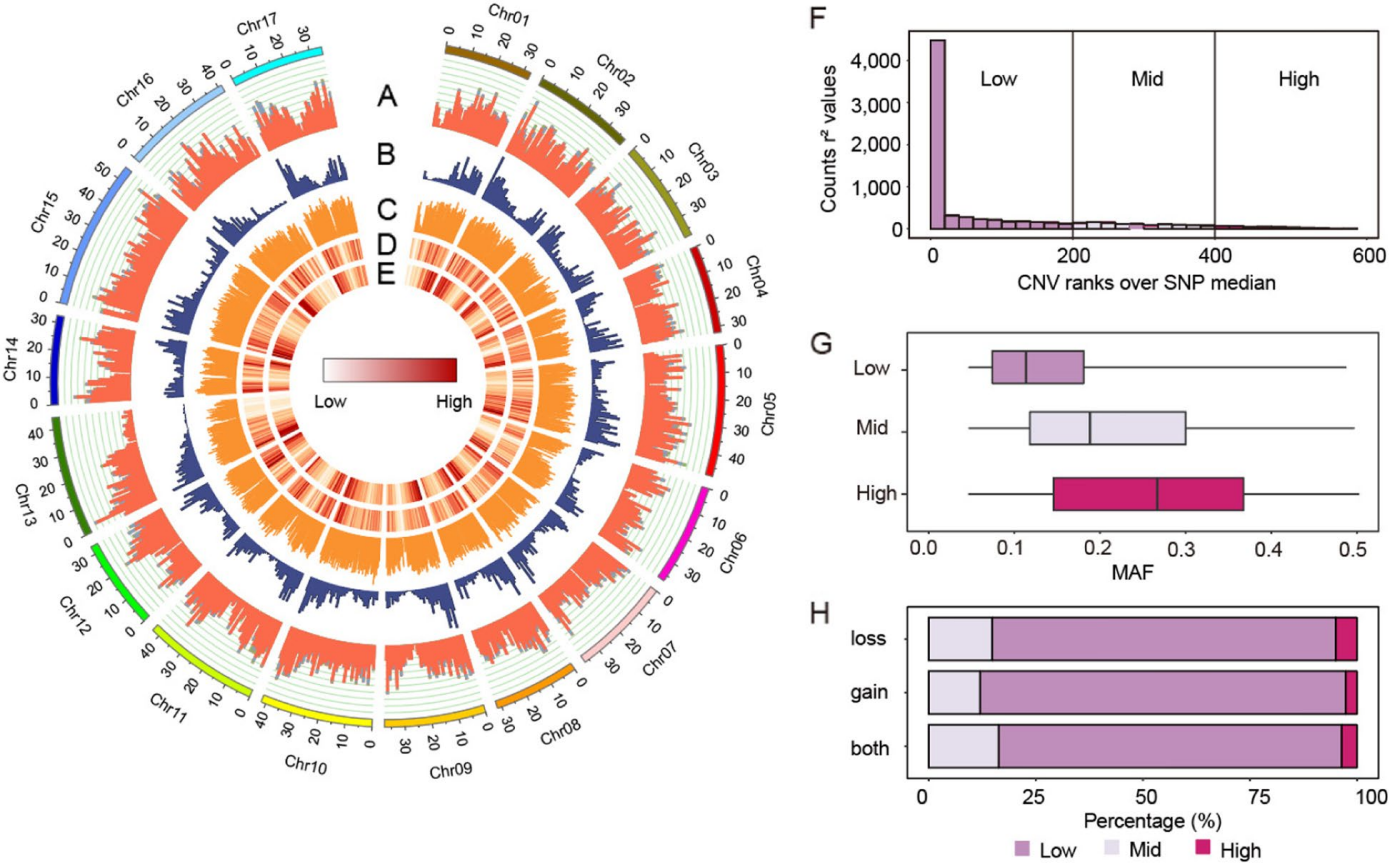
Chromosomal distribution of CNVRs, SNPs and Genes in the apple genome and relationship with SNPs. **A** Numbers of CNVRs including loss (red), gain (blue) and both (green) events in 1-Mb nonoverlapping windows. **B** SNP density (SNPs per 1-Mb window). **C** TE density (TEs per 1-Mb window). **D** CNVR density heatmap (CNVRs per 1-Mb window). **E** Gene density heatmap (genes per 1-Mb window). **F** Histogram of the number of CNVR *r*^2^ ranks that are above the SNP-SNP *r*^2^ median value for CNVRs. **G** Boxplots showing distribution of minor allele frequencies for each LD category. **H** Proportion of each LD category in loss, gain and both events

Next, we merged CNVs across different accessions into CNVRs. A total of 14,839 CNVRs covering ~ 71.19 Mb were identified. These CNVRs account for ~ 10.03% of the reference genome. Then, they were classified into three types with respect to the reference genome: 13,579 loss, 1,048 gain and 212 both events. The length of CNVRs varied from 109 bp to 847 kb with a mean of 4.80 kb (Additional file 2: Table S4). Genome-wide distribution of CNVRs on each chromosome, along with SNPs, TEs and gene features was shown in Fig. 2A-E. It seemed to be consistent between the genomic distribution of TEs and CNVRs.

For each CNVR, the nearby flanking 300 SNPs upstream and downstream were considered for linkage disequilibrium (LD) analysis. CNVRs were then grouped into three categories, Low-LD, Mid-LD and High-LD as illustrated in Methods. A great majority of CNVRs (80%) showed low LD with flanking SNPs, and 15% of CNVRs had intermediate levels of LD, while only 5% exhibited high LD and could be considered tagged by adjacent SNPs (Fig. 2F). Expectedly, more common alleles were more often in a high-LD state, which displayed a positive correlation between LD state and CNVR MAF (Fig. 2G). In addition, proportion of high-LD, mid-LD and low-LD CNVRs were similar among three CNVR types (Fig. 2H).

### CNVR can accurately reflect population structure

**Fig. 3:**
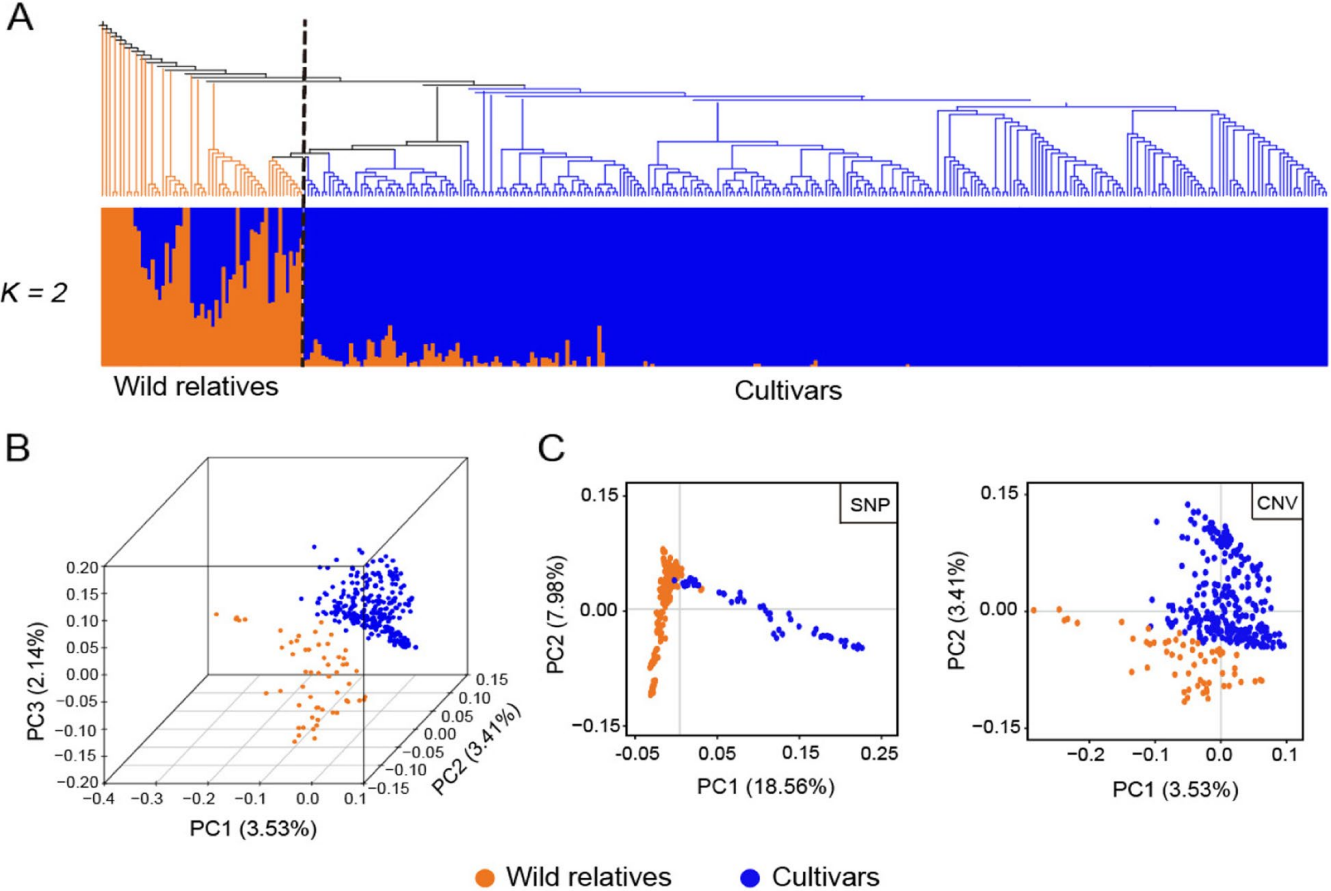
Population genetic analyses of apples based on CNVRs. **A** Clustering results and population structure of 346 apple accessions using CNVRs. Cultivars and wild relatives are represented in blue and orange in the phylogenetic tree, respectively. **B** Principal component analysis of 346 accessions. Accessions are denoted in the same color as in (A). The percentage of variance explained by PC1, PC2, and PC3 are provided. **C** Scatter plots of the first two principal components based on SNPs (left) and CNVRs (right). Accessions are denoted in the same color as in (A). The percentage of variance explained by PC1 and PC2 are provided

Phylogenetic analysis of CNVRs showed that the apple accessions could be separated into cultivar and wild relatives clearly (Fig. 3A, B). Neighbor-joining clustering analysis indicated that *M. sieversii* accessions located close to wild accessions while *M. sylvestris* accessions and cultivars formed a large branch (Additional file 1: Fig. S4).

We then compared our Principal Component Analysis (PCA) results of apple accessions to population structure inferred from SNPs. Although variance explained by PC1 and PC2 of CNVs (6.94%) was lower than SNPs (26.52%), the cultivars and wild relatives could be distinguished in both results (Fig. 3C). While samples in the SNP-based results were more concentrated, the span of samples in CNVR-based was wider for both PC1 and PC2.

### Copy number (CN)-variable genes are mainly enriched in defense and stress response

**Fig. 4:**
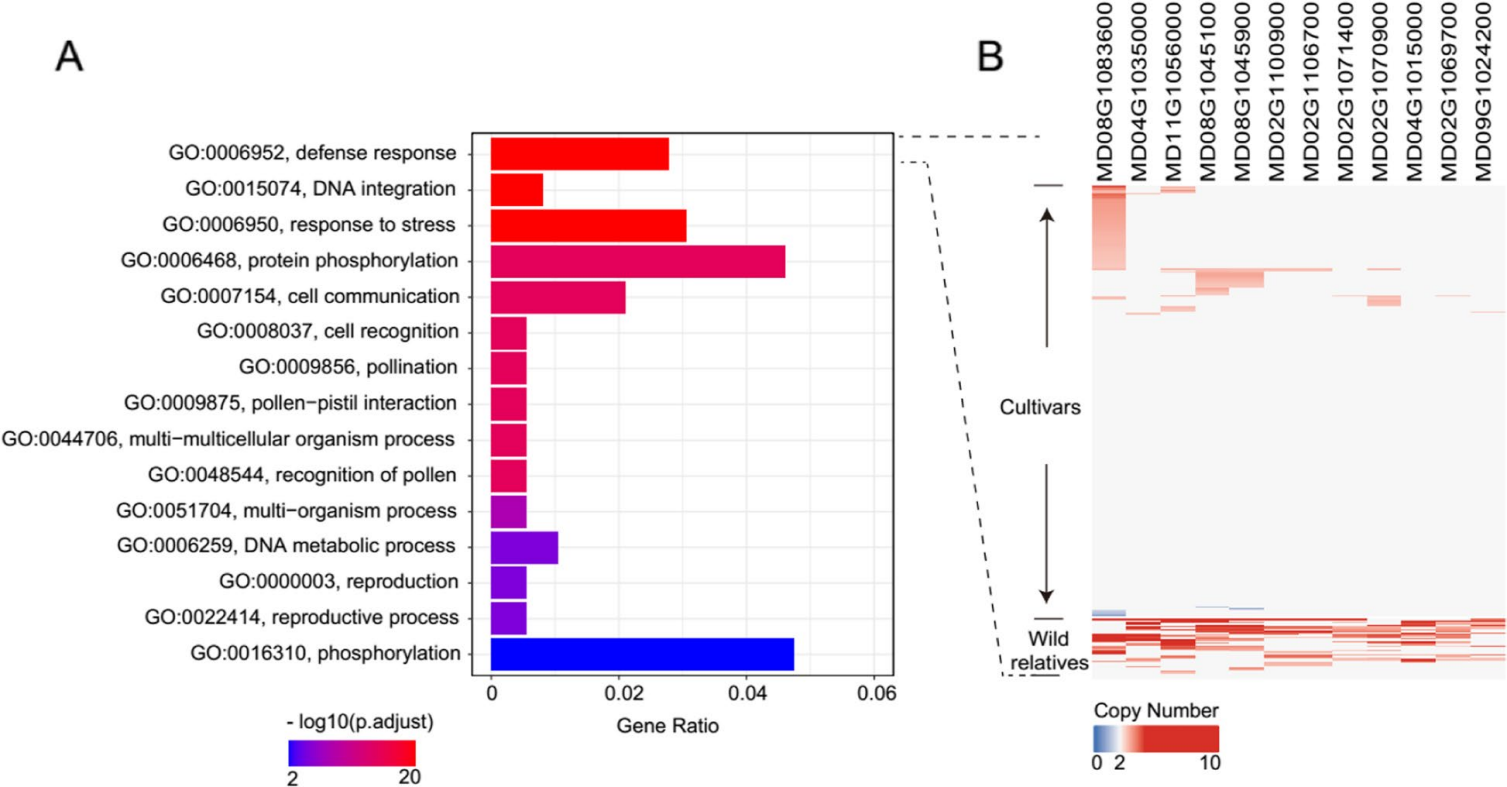
Functional enrichment of CNVR-associated genes. **A** GO analysis of genes affected by CNVRs. **B** CN heatmap of 12 R genes which exhibited significant differences between cultivars and wild relatives

In total, 3,621 genes completely or largely (> 50% of gene span) overlapped with CNVRs were identified in the reference genome (Additional file 2: Table S5). These genes were considered CN-variable genes at the population level. To characterize biological functions of the CN-variable genes, we performed gene ontology (GO) enrichment analysis using the R package topGO [35]. These genes were significantly enriched in defense response (GO:0,006,952, *p* = 5.72 × 10^–20^) and response to stress (GO:0,006,950, *p* = 2.60 × 10^–6^) (Fig. 4A; Additional file 2: Table S6). We also found that 12 R genes (*MD02G1069700*, *MD02G1070900*, *MD02G1071400*, *MD02G1100900*, *MD02G1106700*, *MD04G1015000*, *MD04G1035000*, *MD08G1045100*, *MD08G1045900*, *MD08G1083600*, *MD09G1024200*, and *MD11G1056000*) involved in biological processes such as defense response, response to stress and response to stimulus were CN-variable (Fig. 4B). In addition, many genes were also in connection with cell communication (GO:0,007,154, *p* = 7.28 × 10^–4^), reproduction process (GO:0,022,414, *p* = 0.002), signal transduction (GO:0,007,165, *p* = 9.96 × 10^–3^) and metabolic processes. It is worth noting that several genes involved in the metabolic processes such as malate metabolic process (GO:0,006,108, *p* = 0.020) and sucrose metabolic process (GO:0,005,985, *p* = 0.022) which might be involved in the taste improvement of apple.

### CN-differentiated genes between cultivars and wild relatives

**Fig. 5:**
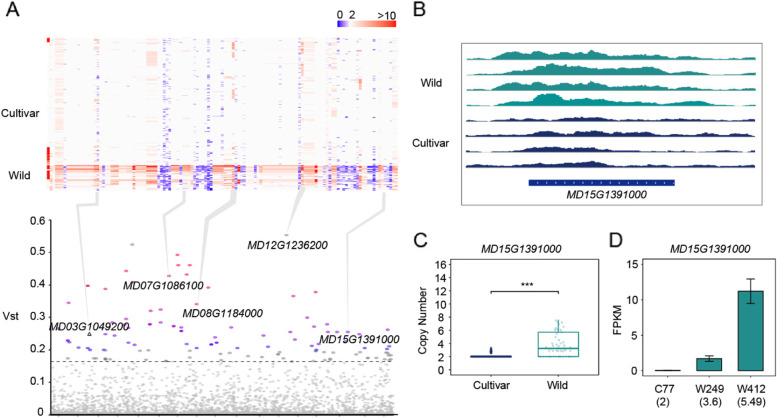
Genes with differentiated CN profiles between cultivars and wild relatives. **A** (Upper) A heatmap of gene CN (columns) for each of the highly differentiated (top1% *V*_st_) genes in 346 accessions (rows). (Lower) A Manhattan plot of values (y axis) for each gene (x axis). The dashed line represents top1% *V*_st_ cutoff. Genes are depicted by their value patterns across the CNVnator CN estimations. **B** The copy number status revealed by genome average around *MD15G1391000* is showed. **C** Copy number status of gene *MD15G1391000* between Cultivars and Wild relatives. *P* value was determined by Wilcoxon rank sum test ***: *p* < 0.001. **D** Expression levels of *MD15G1391000* in three accessions. C77: Cultivars 77; W249: Wild relatives 249; W412: Wild relatives 412. The numbers in the bracket under the accession name are the copy number of the gene in accessions, respectively

To investigate the CN-differentiated genes influenced by CNVs in these two apple populations, gene ${V}_{st}$ between all cultivars and wild relatives (here only *M.sieversii* and *M.sylvestris* were retained) was calculated and genes located in top 1% ${V}_{st}$ were further analyzed (Fig. 5A, B; Additional file 2: Table S7). Within the 127 CN-differentiated genes, 17 were annotated as R genes and most of them harbored higher copy numbers in wild relatives than cultivars (Additional file 1: Fig. S5), which was consistent with the higher disease resistance in wild accessions. For example, *MD03G1049200* (encoding NB-ARC domain-containing disease resistance protein that involved in pathogen recognition and subsequent activation of innate immune responses), has higher CN among wild relatives (mean CN = 3.92) compared with cultivars (mean CN = 2.03) (Additional file 1: Fig. S6 Wilcoxon test; *p* = 9.05 × 10^–12^). Typical resistance genes related to the specific immune response toward pathogen or external adverse stimuli such as *MD15G1391000* also show a similar CN trend (Fig. 5C). We also observed that the expression levels of *MD15G1391000* increased with elevated copy numbers using the expression data of one cultivar and two wild accessions (Fig. 5D).

## Discussion

Over the past decade, SNPs have been commonly used in population genetics-related studies. However, causal loci uncovered by SNPs studies explain only a part of the heritable contribution to trait variation, which results in a phenomenon called as “missing heritability” [36–38]. As an important source of genome variation, CNVs have the potential to fill the gap that SNPs cannot reveal in the population genetics.

In this study, performance of Speedseq was evaluated firstly to determine its availability in CNV detection. Although not detecting all the CNVs, the method keeps extremely low false positive rate (< 1%), which is expected as we aim to identify CNVs with high confidence. The simulation result demonstrates that our strategy is capable to detect majority of CNVs in the data of this work with high accuracy. It was reported that a 625 bp CNV in the 5’UTR of *MdCBF2* which regulates cold acclimation in the GDDH13 reference genome when compared with HFTH1 genome [39]. Correspondingly, we identified a 630 bp deletion in the same position among accessions such as Jonathan and McIntosh, compared with the GDDH13 genome which is among the Golden Delicious accessions (Additional file 1: Fig. S2). Manual check on the reads mapping and CNV detection around *MdCBF2* were also conducted to ensure the accuracy of our results, which suggests that our CNV detection strategy is valid and reliable.

These CNVs were then merged to generate 14,839 CNVRs, which comprised 10.03% of the apple reference genome. The relationship between CNVRs and TEs was explored firstly. The Pearson correlation coefficient of genomic density between CNVRs and TEs was 0.6 (an obvious positive correlation), and 95.92% of CNVRs overlapped with TEs, which demonstrates that CNVRs have an enrichment within TEs to some extent. The resulting CNVR map was also compared with SNPs. In contrast to the highly variable distribution of SNPs along chromosomes, the distribution of CNVR is largely flat, exhibiting a decoupling of the two variation types. The density of CNVR and SNP across the whole genome shows a significant positive correlation with local discordance (*r* = 0.58; *p* < 2.2e-16) (Additional file 1: Fig. S3).

To examine whether there is some relation between CNVRs and SNPs, LD analysis was conducted between each CNVR and nearby 600 SNPs. Similar to results reported in other studies, CNVRs are generally in low linkage (i.e. 80% of CNVRs exhibit low LD state) with SNPs [40–42]. Low LD state can be attributed to two reasons. For one thing, LD is affected by allele frequency, which was reflected by positive correlation between CNVR MAF and LD state. Wray et al. confirmed that the difference in allele frequency of the coupled loci would result in a low LD between them [43]. In this analysis, although we kept CNVRs existed in more than ten accessions, almost half of CNVRs is at low allele frequency (MAF ≤ 0.05), whereas MAF of SNPs are all greater than 0.05. The unmatched allele frequencies of CNVRs and SNPs quite possibly contributed to the low LD. For another thing, SNP density within local regions can explain low LD. As depicted by Redon et al., CNVs were enriched within segmentally duplicated regions of the genome, in which there is a paucity of SNPs [40]. Cooper et al. and McCarroll et al. used different SNP sets in their human CNV analyses, resulting in dissimilar LD state between CNVs and SNPs [44, 45]. In our study, there is a mild discordance in the density of SNPs and CNVRs as plotted in Fig. S3, which may also influence the low LD state. A follow-up study is needed to thoroughly clarify the relationship between CNVs and SNPs. Taken together, as a type of genetic variation that has not been comprehensively characterized in apple, CNVs have potential to associate with important traits that are independent of SNPs.

Population genetics analysis based on CNVRs in apple was the first attempt to investigate relationships among different apple accessions to date. Population structure was largely reflected by dendrogram inferred from both CNVs and SNPs. Recent introgression from *M. sylvestris* into *M.* × *domestica* has been so intensive that cultivars now appear to be closer to *M. sylvestris* than to their progenitor *M. sieversii*. The first two principal components (PCs) based on SNPs explained 26.52% of total variance, and the population was highly structured across PC1. Compared with SNPs, the first two PCs based on CNVs explained less variance (6.94%) while the population was separated clearly along PC2. This result uncovers accessibility of CNVs in population structure inference and underlying information that may not captured by SNPs, which demonstrates significance of CNV in genome variation studies [1, 4].

To better understand the roles of CNVs play, we focus on genes overlapped with CNVRs and examined their GO enrichment. These genes are mainly enriched in defense response and response to stress, which is consistent with a previous study [31]. Among these genes, 211 (5.8%) are annotated as genes encoding proteins with nucleotide binding sites (NBS or NBC-ARC domains) and leucine-rich repeat (LRR) domains as well as genes encoding receptor-like protein kinases (RLK) which are known to be involved in plant defense-related mechanisms [46, 47]. It is worth noting that several genes involved in the metabolic processes such as malate metabolic process (GO:0,006,108, *p* = 0.020) and sucrose metabolic process (GO:0,005,985, *p* = 0.022) which might be involved in the taste improvement of apple. For instance, *MD04G1101300* and *MD17G1077700* were malate degradation related genes encoding NADP-malic enzyme (NADP-ME). The rapid decrease of malate content during fruit ripening has already been attributed to its degradation by cytosolic NADP-ME in some fruits such as loquat [48]. The high-level expression of NADP-ME increased the ratio of total sugars to total acids by regulating the fruit acidity in apple, leading to the improvement of fruit taste and sensory evaluation [49]. *MD03G1248900*, a gene encoding NAD(H) kinase 3, homolog of NADK3 whose up-regulation of expression coincided with fruit growth at ripening stages in tomato, might also involve in the enlargement process in the apple fruit [50].

During the process of plants domestication, CNVs are found to be related to stress tolerance, disease resistance and development [6]. Considering that cultivated apples were domesticated from *M.sieversii* and *M.sylvestris*, CN-differentiated were identified between cultivars and wild relatives, which only consisted of *M.sieversii* and *M.sylvestris*. Among 127 CN-differentiated genes, 17 of them are annotated as R genes and exhibit higher copy numbers in wild relatives than cultivars, which is consistent with the higher disease resistance in wild accessions. Higher copy number might play key roles in stronger stress resistance in wild relatives compared to cultivars. The most prominent gene is *MD12G1236200* (*XRN4*), which was at higher CN among wild relatives (mean CN = 3.06) compared with cultivars (mean CN = 2.00) (Wilcoxon test; *p* = 6.59 × 10^–44^)*.* It was reported that *XRN4* involved in ethylene response and disease resistance in Arabidopsis [51]. The decreased CN of *MD12G1236200* might also be related to the changes of similar function, which needs to be verified by wet experiments. Besides, several CN-variable genes appear to be involved in fruit developmental processes (Additional file 1: Fig S6). *MD08G1184000*, designated as *MdGF14f*, has an abnormal copy number in some wild relatives (mean CN = 1.14) while almost no changes were observed in cultivars (mean CN = 2.00) (Wilcoxon test; *p* = 3.04 × 10^–15^). High expression in flowers indicates that it may play an important role in apple growth and development [52]. *MD07G1086100* belongs to MADS-box gene family. Sequence analysis from appleMDO database (http://bioinformatics.cau.edu.cn/AppleMDO/) indicated that *MdMADS110* was ubiquitously expressed in different tissues including apical bud, spur bud and flower [53, 54]. Studies have revealed that it involved in plant reproduction processes [52]. This analysis indicated that *MD07G1086100* showing differentiated CN is likely to play possible regulatory roles in apple development. The decrease of gene copy numbers might be relevant to potential adaptability or neutrality of non-functionalization mutations, which supports the ‘less is more’ or ‘regression evolution’ hypothesis [55].

## Conclusions

In this study, we carried out comprehensive CNV analyses in wild and cultivated apples. In total, 14,839 CNVRs occupying 10.03% of the apple genome were identified, and a comprehensive map of CNVs was constructed based on 346 apple accessions. The low LD between CNVRs and SNPs indicates that CNVs are largely independent genetic variation resources. Genes overlapping with CNVRs were mainly enriched in defense response, reproduction and other metabolic processes. Some genes overlapping CNVRs are highly differentiated between cultivars and wild relatives, which appears to be related with some differences in traits between them. To confirm their genetic associations, functional verification is required in follow-up studies. These findings will provide important resources for comprehensive understanding of the genome variation and may serve as a useful reference for future genomic studies on apple and related species.

## Methods

### CNV simulation

To test the performance of the method used in this study, 200 duplications and 200 deletions were simulated in apple genome using the RSVSim (v1.28.0), an R package that can be used to simulate CNVs across a genome [56]. The simulated reads were generated from the modified genome using wgsim (v1.9; https://github.com/lh3/wgsim). It was noting that we simulated reads at various levels of sequencing coverage ranging from 5 to 30X as we aimed to investigate the relationship between discovery power and coverage depth in CNV detection. Next, the reads were mapped to the unmodified apple genome and used for CNV detection following subsequent calling analysis. The true positive, false positive and false negative CNV discovery rates were calculated to evaluate performance of the method.

### Samples and NGS data processing

A total of 346 accessions which have clear pedigree information were used for CNV detection. This dataset consists of 289 cultivars and 57 wild relatives. The corresponding re-sequencing data was obtained from previous studies with accession number CRA003964 in the Genome Sequence Archive (GSA) database and SRP075497 in the NCBI Sequence Read Archive [4, 32]. The reference genome GDDH13 and gene annotation files were downloaded from the Genome Database for Rosaceae (GDR) (https://www.rosaceae.org/species/malus/malus_x_domestica/genome_GDDH13_v1.1) [57]. Raw reads were preprocessed to remove low-quality reads and adapter sequences by Trimmomatic (v0.38) with the following parameters: SLIDINGWINDOW, 4:15; TRAILING, 20; HEADCROP, 3; MINLEN, 90 [58]. Clean reads were then mapped to the apple reference genome using BWA-MEM (v0.7.17) with default parameters and SAM format files were sorted and indexed into BAM format files using Samtools (v1.5) [59, 60]. Potential duplicate reads generated in PCR amplification were removed using Picard (v2.17.0) (http://broadinstitute.github.io/picard/). The BAM files were further filtered based on mapping quality (reads with MAPQ < 20 were discarded) for subsequent analysis [31].

Three available RNA-seq datasets corresponding to accessions with sample number C77, W249 and W412 (C77 belongs to cultivars, W249 and W412 belong to wild relatives) was obtained from GSA database (CRA003991) under accession number CRR274993, CRR274994, CRR274995, CRR274999, CRR275000, CRR275001, CRR275002, CRR275003, CRR275004. Each sample consisted of three biological replicates. We processed these data as described previously [32] and used the average FPKM of three replicates to measure abundance of gene expression.

### CNV calling and CNVR definition

SpeedSeq, a software package that integrates RP, SR and RD method, was used for CNV detection in this analysis [26]. It incorporates Lumpy (v0.2.13) and CNVnator (v0.3) to detect CNV together [33, 34]. In detail, Lumpy with the default parameters was run in the Lumpy Express script as well as the -P option to output probability curves for each breakpoint on each accession. Then CNVnator was used to annotate the copy number of each variant using the sliding window of 1 kb and filtering the raw calls with a cutoff *P*-value of 0.05. Finally, only deletions and duplications supported by RD, SR and RP analysis simultaneously and longer than 50 bp were retained for further analysis.

The CNV region (CNVR) is defined as a combined region of overlapping CNVs in the apple genome after aggregating and filtering out the ones existed in less than ten accessions. Here, CNVR were merged from different accessions with at least 90% stringently reciprocal overlap by extending the boundaries of the overlapping CNVs. Then they were further classified as “gain” (i.e. CNVRs merged from duplications across different accessions), “loss” (i.e. CNVRs merged from deletions across different accessions) and “both” (i.e. CNVRs merged from both duplications and deletions within the same regions across different accessions) events according to their types. Only CNVR presented in at least ten individuals were used for subsequent functional and population genetics analysis, thus minimizing the bias caused by uniformity of sequence coverage depth and impact of rare CNVR.

In order to facilitate subsequent analysis, all CNVRs were recoded manually by converting a loss event into “0/1”, a neutral event into “0/0” and a gain event into “1/1”, thus we converted the file into VCF-format file and filtered the CNVRs with MAF < 0.05 to keep consistent with criterion used in SNPs filtration for comparison with SNPs in population structure analysis.

### Relationship analysis between CNVs and SNPs

Transposable element (TE) was shown as a source of novel genetic diversity in Arabidopsis because of large extent not in linkage disequilibrium (LD) with nearby SNPs [61]. As TE activity is an important source of CNV formation, the relationship between CNVs and SNPs in apple was then investigated to observe whether there are similar phenomena as described in Arabidopsis [61]. SNP information for apple accessions was obtained from BIG Data Center (https://bigd.big.ac.cn/) with accession number GVM000128. Accessions with both CNV and SNP information were selected for analysis, thus 343 samples were retained. For each CNVR, the nearest 300 upstream and 300 downstream SNPs with a minor allele frequency greater than 5% were selected. Pairwise genotype correlations (*r*^2^ values) for all SNP-SNP and SNP-CNVR pairs were calculated by PLINK (v1.90p) [62]. Then, *r*^2^ values ordered by decreasing rank and a median SNP-SNP rank value was calculated. According to the number of times (*N*) CNVR rank over the SNP-SNP median rank, each CNVR was classified as Low-LD, Mid-LD and High-LD to SNP as follows:

$$\begin{aligned} \text{LD}=\left\{\begin{array}{cc} {\text{Low}}& {0} \, \, \leq {\text{N}} \, \leq \, {200} \, \text{;}\\ {\text{Mid}} & {200} \, < \, {\text{N}} \, \leq \, {400} \, \text{;}\\ {\text{High}}& {400} \, < \, {\text{N}} \, \leq \, {600}\end{array}\right. \end{aligned}$$

### Population-genetic properties derived from apple CNVRs

To explore the population structure and convergence of accessions, admixture analysis was performed ADMIXTURE (v1.3.0) with 500 replicates and cross-validation error (CV) procedure was run [63]. Neighbor-joining clustering analysis based on pairwise genetic distance matrices was conducted using PHYLIP (v3.697) with 1,000 bootstrap replicates [64] and the clustering dendrograms were visualized in iTOL [62, 65]. Principal component analysis (PCA) was performed using the smartpca program of the Eigensoft software package (v6.0.1) [66].

### Gene annotation and enrichment analysis

Gene contents encompassed by CNVR were assessed by comparing coordinates between them. Only genes covered by CNVR more than 50% were considered for functional analysis. GO enrichment analysis was carried out on these genes using the R package topGO (v2.28.0) [35]. Significance of GO terms were determined using Fisher's exact test with FDR correction, and GO terms were considered significant if the adjusted *P*-value was below 0.05.

### CN-differentiated genes between cultivars and wild relatives

Because cultivars have been domesticated mainly from *M. sieversii* and *M. sylvestris*, only wild relatives belonging to these two species were considered in this analysis [1]. The statistic ${\text{V}}_{\text{st}}$ was used to identify divergent genes between the cultivars and wild relatives. It was devised specially to measure the population differentiation at CNV levels and varies from 0 to 1 representing no differentiation and complete differentiation respectively [40]. ${\text{V}}_{\text{st}}$ was calculated as follows:

$${\text{V}}_{\text{st}} {=} \frac{\left({\text{V}}_{\text{total}} -\left({\text{V}}_{{\text{cultivars}}^{\times }}{\text{N}}_{\text{cultivars}}{+}{\text{V}}_{{\text{wild relatives}}^{\times }}{\text{N}}_{\text{wild relatives}}\right)\right)/{\text{N}}_{\text{total}}}{{\text{V}}_{\text{total}}}$$

where ${\text{V}}_{\text{total}}$ is total variance in copy number measured by CNVnator among all apple accessions, ${\text{V}}_{\text{cultivars}}$ is copy number variance among all cultivars and ${\text{V}}_{\text{wild relatives}}$ is copy number variance among all wild relatives. ${\text{N}}_{\text{cultivars}}$ and ${\text{N}}_{\text{wild relatives}}$ is the sample size for the cultivars and wild relatives, respectively; ${\text{N}}_{\text{total}}$ is the total sample size. Thus ${\text{V}}_{\text{st}}$ was calculated across all genes using CN estimates obtained from CNVnator.

## Supplementary Information

**Additional file 1: Fig. S1.** Test of the Speedseq using simulated CNVs in the GDDH13 genome, for a range of sequencing coverage levels. Deletions (A) and duplications (B). TP, True positives; FN, False negatives; FP, False positives.**Fig. S2.** Examples of CNVs identified. (Upper) A 630bp deletion in the 5’UTR of MdCBF2 (MD01G1196100). (Lower) MD15G139100 was fully overlapped by a duplication on the Chromosome 15. **Fig. S3.** SNP versus CNV densities in 1Mb sliding windows with 500kb step size across the genome. The local discordance was marked with a red circle.** Fig. S4.** Neighbor-joining clustering for all accessions with each color of branch corresponds to a single pedigree as noted.** Fig. S5.** 17 R genes with differentiated CN profiles (top 1% Vst) between cultivars and wild relatives.** Fig. S6.** Boxplot of CN profiles of genes related with various biological functions. Whiskers extend to the highest and lowest values no greater than 1.5 times the inner quartile range. For both comparisons, groups were significantly different - Wilcoxon rank sum test with continuity correction, *p* < 0.0001.

**Additional file 2: TableS1.** Summary of 346 apple accessions and resequencing data used in the CNV analysis. **Table S2.** Summary of CNV events in different accessions. **Table S3.** Extent of apple genome features impacted by non-overlapping CNVs. **Table S4.** List of all CNVRs and CN estimated by CNVnator in the apple genomes. **Table S5.** The genes completely or partially inside (50% overlap) of the identified CNVRs in the apple genome. **Table S6.** GO Biological Process enrichment for all CNVR genes. **Table S7.** Population-differentiated CNV-Genes between cultivars and wild relatives. (Here, wild relatives only consist of M.sieversii and M.sylvestris).

## Data Availability

The whole genome re-sequencing data was obtained from Genome Sequence Archive (GSA) database with accession number CRA003964 and NCBI Sequence Read Archive (SRA) database with accession number SRP075497. RNA-seq datasets were obtained from GSA database with accession number CRA003991. SNP information was obtained from BIG Data Center (https://bigd.big.ac.cn/) with accession number GVM000128. The reference genome GDDH13 version 1.1 and corresponding gene annotation files were downloaded from the Genome Database for Rosaceae (GDR) (https://www.rosaceae.org/species/malus/malus_x_domestica/genome_GDDH13_v1.1). All data analyzed in this study are included in this article and its Additional files.

## Abbreviations

CN: Copy number
CNV: Copy number variation
CNVR: Copy number variation region
SNP: Single nucleotide polymorphism
TE: Transposable element
RP: Read-pair
SR: Split-read
RD: Read-depth
aCGH: Array comparative genomic hybridization
NGS: Next-generation sequencing
TPR: True positive rate
LD: Linkage disequilibrium
PCA: Principal component analysis
GO: Gene ontology
NBS: Nucleotide binding sites
LRR: Leucine-rich repeat
RLK: Receptor-like protein kinases
NADP-ME: NADP-malic enzyme
VLCFA: Very long chain fatty acids
