# Supplementary material for: A comprehensive analysis of copy number variations in diverse apple populations
Source: BMC Genomics. 2023 May 11;24:256. doi: 10.1186/s12864-023-09347-9 (PMC10176694; doi:10.1186/s12864-023-09347-9)
Supplement: Supplementary file 1 — Additional file 1: Fig. S1. Test of the Speedseq using simulated CNVs in the GDDH13 genome, for a range of sequencing coverage levels. Deletions (A) and duplications (B). TP, True positives; FN, False negatives; FP, False positives.Fig. S2. Examples of CNVs identified. (Upper) A 630bp deletion in the 5’UTR of MdCBF2 (MD01G1196100). (Lower) MD15G139100 was fully overlapped by a duplication on the Chromosome 15. Fig. S3. SNP versus CNV densities in 1Mb sliding windows with 500kb step size across the genome. The local discordance was marked with a red circle. Fig. S4. Neighbor-joining clustering for all accessions with each color of branch corresponds to a single pedigree as noted. Fig. S5. 17 R genes with differentiated CN profiles (top 1% Vst) between cultivars and wild relatives. Fig. S6. Boxplot of CN profiles of genes related with various biological functions. Whiskers extend to the highest and lowest values no greater than 1.5 times the inner quartile range. For both comparisons, groups were significantly different - Wilcoxon rank sum test with continuity correction, p < 0.0001. [file 12864_2023_9347_MOESM1_ESM.docx]

A comprehensive analysis of copy number variations in diverse apple populations

Jinsheng Xu^1,#^, Weihan Zhang^1,#^, Ping Zhang^1^,Weicheng Sun^1^, Yuepeng Han^2,3,*^, Li Li ^1,3,*^

^1^Hubei Key Laboratory of Agricultural Bioinformatics, College of Informatics, Huazhong Agricultural University, Wuhan 430070, China

^2^CAS Key Laboratory of Plant Germplasm Enhancement and Specialty Agriculture, Wuhan Botanical Garden, The Innovative Academy of Seed Design, Chinese Academy of Sciences, Wuhan 430074, China

^3^Hubei Hongshan Laboratory, Huazhong Agricultural University, Wuhan 430074, China

^#^ Contribute equally

*Corresponding author

E-mail addresses:

Jinsheng Xu: [jsxu@webmail.hzau.edu.cn](mailto:%20jsxu@webmail.hzau.edu.cn)

Weihan Zhang: [whzhang@webmail.hzau.edu.cn](mailto:whzhang@webmail.hzau.edu.cn)

Ping Zhang: [pingzhang@webmail.hzau.edu.cn](mailto:pingzhang@webmail.hzau.edu.cn)

Weicheng Sun: [weichengsun@webmail.hzau.edu.cn](mailto:weichengsun@webmail.hzau.edu.cn)

Yuepeng Han: yphan@wbgcas.cn

Li Li: [li.li@mail.hzau.edu.cn](mailto:li.li@mail.hzau.edu.cn)


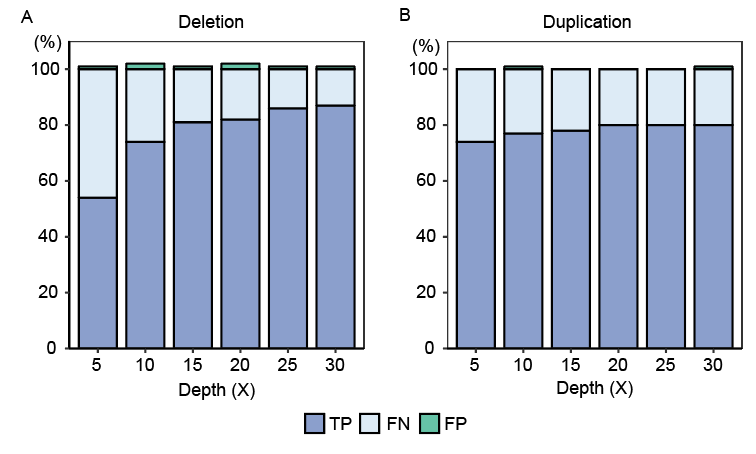


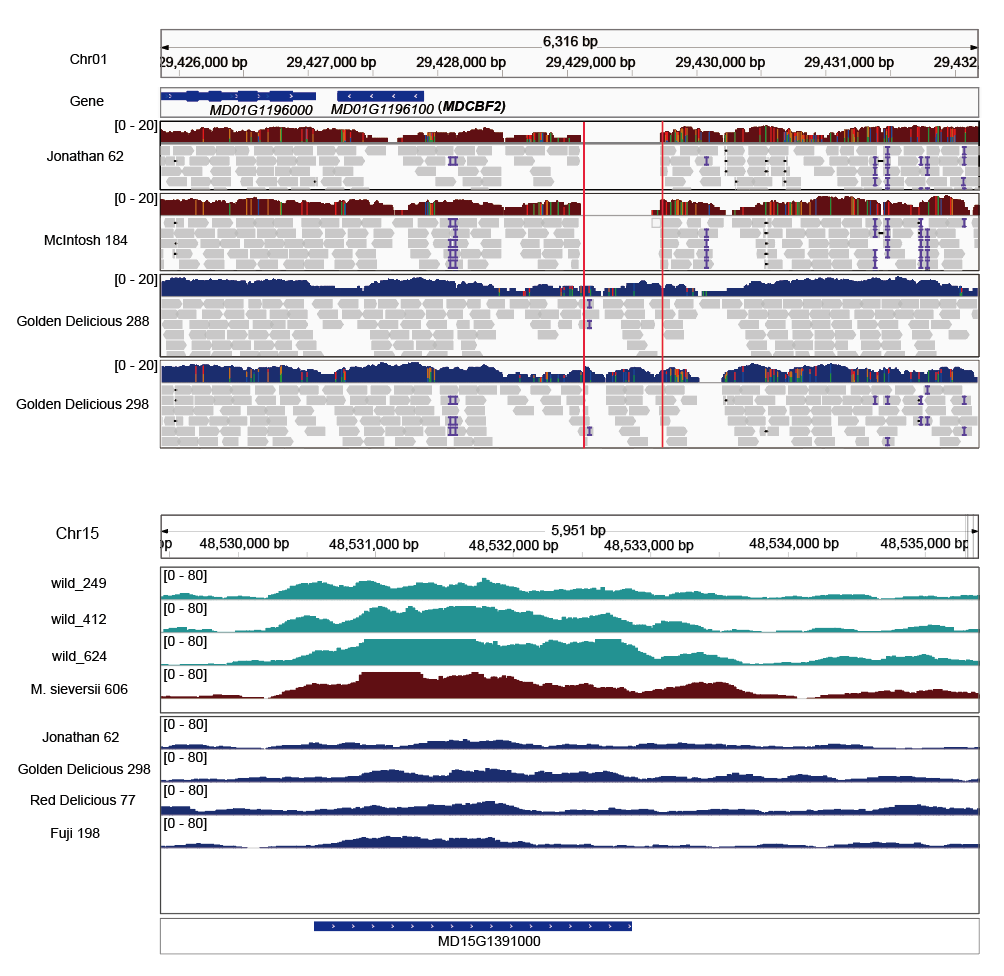
Fig. S1. Test of the Speedseq using simulated CNVs in the GDDH13 genome, for a range of sequencing coverage levels. Deletions (A) and duplications (B). TP, True positives; FN, False negatives; FP, False positives.

Fig. S2. Examples of CNVs identified. (Upper) A 630bp deletion in the 5’UTR of *MdCBF2* (*MD01G1196100*). (Lower) *MD15G139100* was fully overlapped by a duplication on the Chromosome 15.


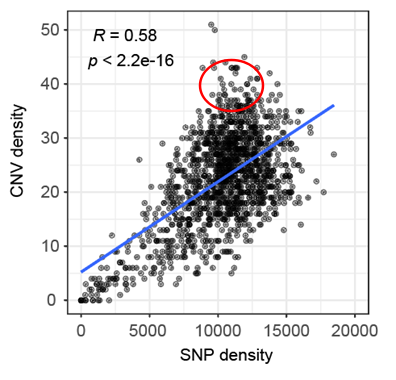


Fig. S3. SNP versus CNV densities in 1Mb sliding windows with 500kb step size across the genome. The local discordance was marked with a red circle.


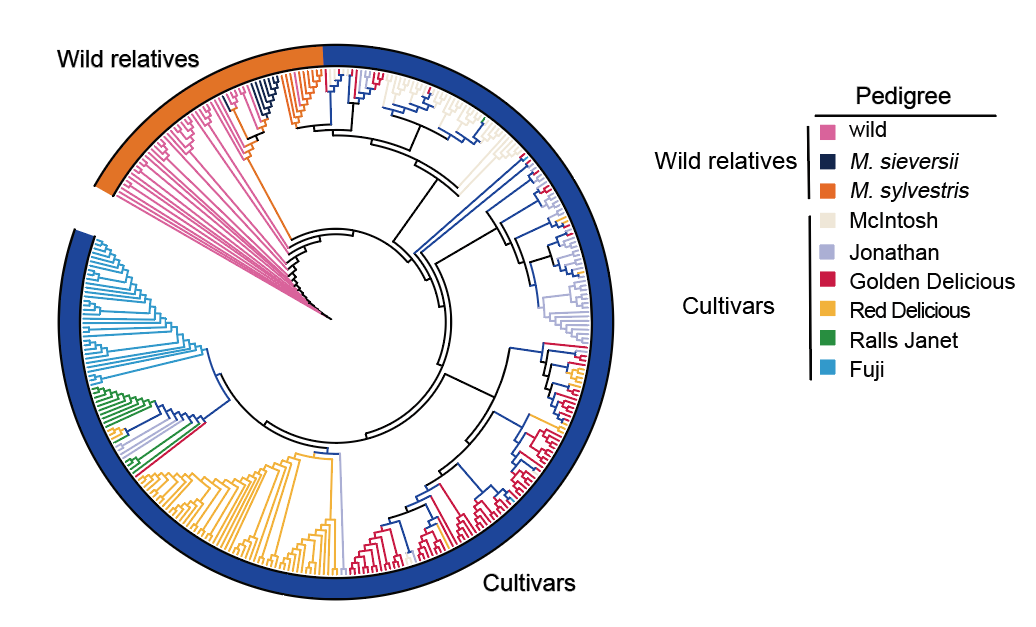


Fig. S4. Neighbor-joining clustering for all accessions with each color of branch corresponds to a single pedigree as noted.

Fig. S5. 17 R genes with differentiated CN profiles (top 1% *V*_st_) between cultivars and wild relatives.


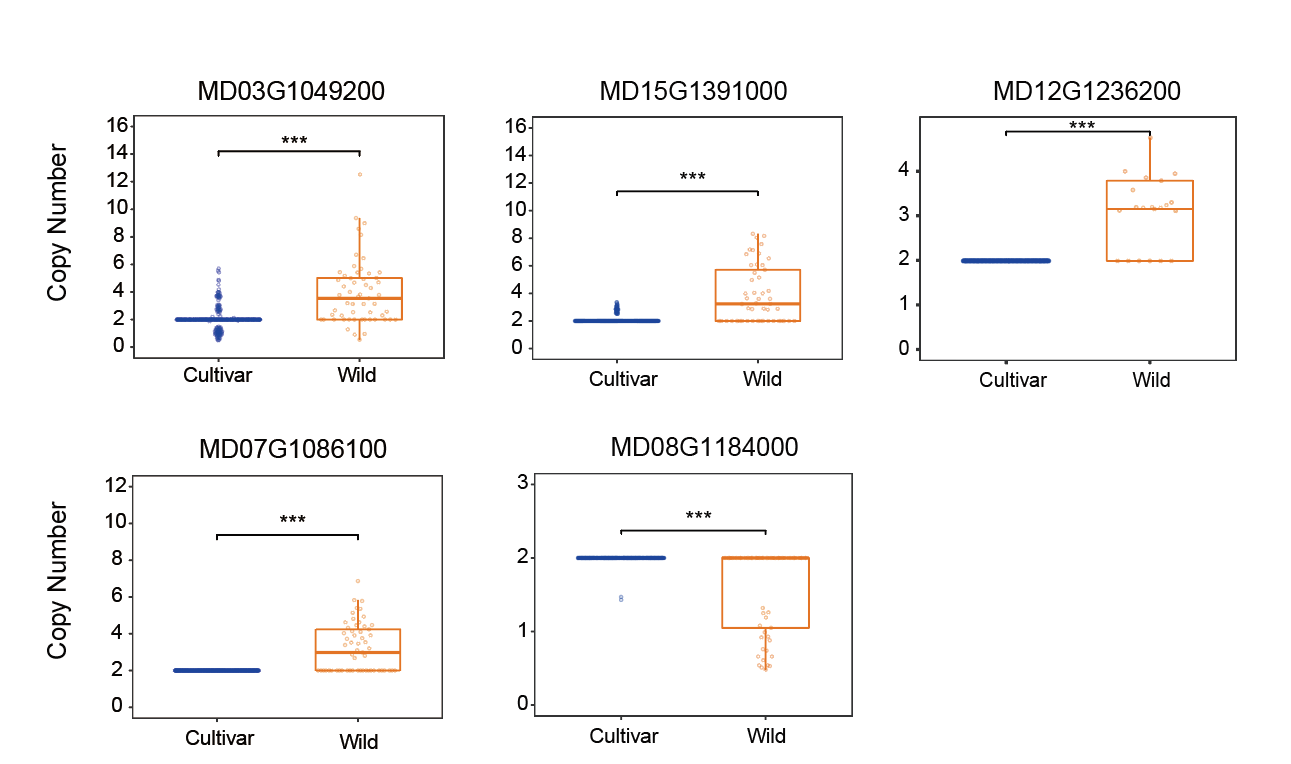

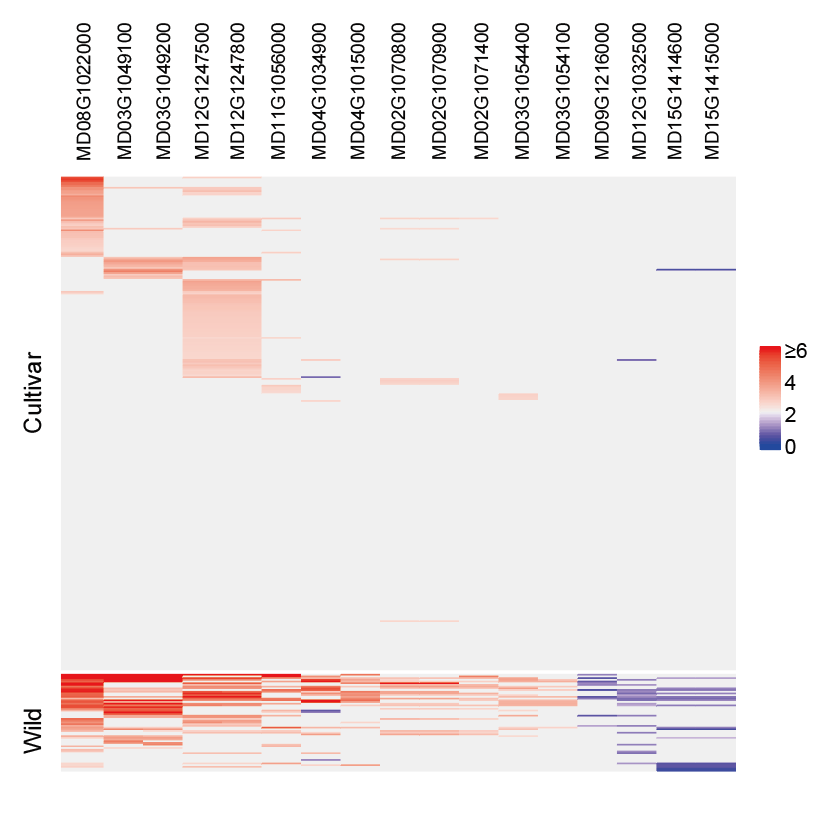


Fig. S6. Boxplot of CN profiles of genes related with various biological functions. Whiskers extend to the highest and lowest values no greater than 1.5 times the inner quartile range. For both comparisons, groups were significantly different - Wilcoxon rank sum test with continuity correction, *p* < 0.0001.
